# Bisphosphonate treatment of aggressive primary, recurrent and metastatic Giant Cell Tumour of Bone

**DOI:** 10.1186/1471-2407-10-462

**Published:** 2010-08-29

**Authors:** Maurice Balke, Laura Campanacci, Carsten Gebert, Piero Picci, Max Gibbons, Richard Taylor, Pancras Hogendoorn, Judith Kroep, John Wass, Nicholas Athanasou

**Affiliations:** 1 Department of Orthopaedic Surgery, University of Munster, Albert-Schweitzer-Str. 3, 48149 Munster, Germany; 2 Laboratory of Oncologic Research, Orthopaedic Institute Rizzoli, Via di Barbiano 1/10, 40136 Bologna, Italy; 3 University of Oxford, Nuffield Department of Orthopaedic Surgery, Department of Pathology, Nuffield Orthopaedic Centre, Oxford, OX3 7LD, UK; 4 Department of Pathology, Leiden University Medical Centre, Albinusdreef 2, Leiden, P.O. Box 9600, L1-Q, 2300 RC Leiden, The Netherlands

## Abstract

**Background:**

Giant cell tumour of bone (GCTB) is an expansile osteolytic tumour which contains numerous osteoclast-like giant cells. GCTB frequently recurs and can produce metastatic lesions in the lungs. Bisphosphonates are anti-resorptive drugs which act mainly on osteoclasts.

**Method:**

In this study, we have examined clinical and radiological outcomes of treatment with aminobisphosphonates on 25 cases of aggressive primary, recurrent and metastatic GCTB derived from four European centres. We also analysed in vitro the inhibitory effect of zoledronic acid on osteoclasts isolated from GCTBs.

**Results:**

Treatment protocols differed with several different aminobisphosphonates being employed, but stabilisation of disease was achieved in most of these cases which were refractory to conventional treatment. Most inoperable sacral/pelvic tumours did not increase in size and no further recurrence was seen in GCTBs that had repeatedly recurred in bone and soft tissues. Lung metastases did not increase in size or number following treatment. Zoledronic acid markedly inhibited lacunar resorption by GCTB-derived osteoclasts in vitro.

**Conclusion:**

Our findings suggest that bisphosphonates may be useful in controlling disease progression in GCTB and that these agents directly inhibit GCTB - derived osteoclast resorption. These studies highlight the need for the establishment of standardised protocols to assess the efficacy of bisphosphonate treatment of GCTB.

## Background

Giant cell tumour of bone (GCTB) accounts for around 5% of all primary bone tumours. GCTB is an expansile osteolytic tumour which most often arises at the end of a long bone in a skeletally mature patient [1,2]. Clinically, most GCTB patients present with bone pain due to enlargement of the tumour. GCTB is characterised by the presence of numerous giant cells that exhibit the phenotypic features of mature osteoclasts [3]. The functional definition of an osteoclast is that it is capable of resorbing bone and, not surprisingly, GCTB is a tumour that causes extensive osteolysis. GCTBs are actively growing tumours which are often locally aggressive, eroding the bone cortex and extending into surrounding soft tissues [1,2]. Metastatic lesions, most commonly in the lungs, can also develop in approximately 2% of GCTB cases. The treatment of primary GCTBs is essentially surgical and includes curettage (with or without adjuvants such as phenol or liquid nitrogen) and excision of the affected area; however, surgical excision and reconstruction is difficult as the tumour commonly involves the epiphysis and the tumour arises in relatively young patients, thus limiting surgical options. Reported recurrence rates after curettage vary, depending upon the thoroughness of curettage and the adjuvant treatment employed, but are generally in the range of 25 to 50% [4-7].

Bisphosphonates are widely used to inhibit osteolysis in conditions such as osteoporosis, Paget's disease and metastatic cancer; these agents act by inhibiting osteoclastic bone resorption by a variety of means [8-10]. Bisphosphonates have been used to treat osteolytic benign tumour-like lesions such as fibrous dysplasia and Langerhans cell histiocytosis that are not amenable to surgery [11-14]. There are also a few reports of aminobisphosphonate treatment of primary GCTBs which have shown a variable but generally beneficial effect on tumour size and recurrence rate [15,16]. There are no reports, however, on the effect of bisphosphonate treatment on recurrent or metastatic GCTBs.

Tumour osteolysis is mediated by osteoclasts [17,18], and GCTB is a tumour that contains numerous osteoclasts. Bisphosphonates act primarily by inhibiting osteoclast-mediated resorption and it would seem logical that GCTBs would benefit from bisphosphonate therapy. A number of major European bone tumour centres have tried using aminobisphosphonates to control disease progression in problematic GCTB cases. Included amongst these cases were persistently recurrent GCTBs and large, pelvic or sacral GCTBs that were deemed inoperable on account of difficulty in obtaining surgical clearance, as well as a number of GCTB cases with evidence of metastatic disease. The aim of this study is to report the outcome of aminobisphosphonate treatment of these GCTBs. We have also analysed the effect of zoledronic acid (one of the most commonly employed bisphosphonates) on lacunar resorption by osteoclasts isolated directly from GCTBs in order to provide in vitro evidence for the inhibitory effect of aminobisphosphonates on GCTB osteolysis.

## Methods

### Clinical studies: Aminobisphosphonate-treatment of GCTB cases

25 GCTB cases which had received aminobisphosphonate treatment were recruited from partners in the EuroBoNet consortium. The bisphosphonate treatment protocol was approved by the ethics review board of each participating institution. The study was carried out in accordance with the ethical principles of the Declaration of Helsinki and good clinical practice. All patients provided written informed consent before treatment. 11 cases were obtained from the Istituti Ortopedici Rizzoli, Bologna, 4 from The Nuffield Orthopaedic Centre, Oxford, 7 from Westfalische Wilhmsuniversitai, Munster, and 4 from Leiden University Medical Centre, Leiden. Clinical details of these bisphosphonate-treated cases, including sex, age, site of tumour, episodes of tumour recurrence following surgery and evidence of metastasis, are shown in Table 1. All cases included in this study were Enneking Stage 1B or 3B.

**Table 1:** Details of bisphosphonate-treated cases

Sex/Age	Tumour Site	Pre treatment Recurrence/Metastasis	Post treatment Follow-up (months)	Type of bisphosphonate (and other) treatment employed	Post treatment Radiological/Clinical Outcome
1. M 39	Distal femur	RRR/-	24	Alendronate	Pain decrease No further recurrence
2. F 28	Sacrum	*/-	36	Clodronate (Interferon α, Radiation)	Pain decrease Stable size
3. M 63	Distal femur	R/M	12	Clodronate (Interferon α)	No further recurrence Lung mets stable
4. M 16	Distal Femur, Proximal Femur, Proximal Tibia (Multifocal)	R/M	60	Clodronate (Interferon α, Cis-platin, Adriamycin)	Stable size Lung mets stable
5. M 20	Proximal Femur	RR/M	64	Pamidronate (Interferon α)	No further recurrence Lung mets stable
6. M 47	Pelvis	R/-	50	Clodronate (Radiation)	Stable size
7. F 56	Sacrum	R/-	32	Alendronate (Radiation, Embolisation)	Pain decrease Stable size
8. F 75	Sacrum	*/-	24	Zoledronic acid	Stable size
9. F 42	Proximal Femur	RRRRR/-	24	Zoledronic acid	No further recurrence Pain decrease
10. M 34	Vertebra	RR/-	12	Zoledronic acid (Radiotherapy, Embolisation)	Stable size Recurrence
11. M 32	Fibula	RR(ST)/M	62	Zoledronic acid	Pain decrease No further recurrence Lung mets stable
12. F 21	Sacrum	RR/-	60	Zoledronic acid (Embolisation)	Disease progression
13. F 45	Sacrum	M	12	Zoledronic acid (Embolisation)	Decrease in size Pain decrease
14. M 74	Pelvis	*/-	26	Zoledronic acid (Embolisation)	Pain decrease Stable size Paget's
15. F 19	Sacrum	R/-	15	Zoledronic acid (Embolisation)	Stable size No further recurrence
16. F 48	Sacrum	R/-	24	Zoledronic acid ^a ^(Embolisation)	Stable size No further recurrence
17. M 15	Sacrum	R/-	12	Zoledronic acid ^a ^(Embolisation)	Stable size No further recurrence
18. F 22	Distal Femur (multifocal)	RR/M	12	Zoledronic acid	Disease progression
19. F 17	Proximal tibia	R/M	24	Zoledronic acid (Interferon α, Cyclophosphamide, Ifosfamide)	Lung mets stable No further recurrence
20. F 32	Pelvis	R/M	24	Zoledronic acid (Radiation, Embolisation)	Stable size Lung mets stable
21. F 64	Proximal tibia	R/M		Zoledronic acid (Embolisation)	No further recurrence Lung mets stable
22. F 30	Proximal humerus	-/-	24	Zoledronic acid	Pain decrease No further recurrence
23. F 49	Sacrum	R/M	3	Zoledronic acid (Embolisation)	Disease progression
24. F 31	Fibula	(ST)RR/M	30	Zoledronic acid ^a^	Lung mets stable No further recurrence
25. F 51	Distal radius	RR/M	8	Zoledronic acid	Lung mets stable

* No surgical treatment

a - intra-op zoledronic acid for treatment of recurrent tumour

ST - soft tissue recurrence

R - episode of recurrence

M - lung metastasis

Indications for patient referral for bisphosphonate treatment differed between the various centres. Most centres used bisphosphonates for the treatment of recurrent or metastatic disease or for primary inoperable pelvic/sacral tumours. In some cases, additional adjuvant therapy, such as embolisation, radiation therapy or other drugs (eg interferon alpha) were also used to control progressive disease. Zoledronic acid was the most commonly employed bisphosphonate; other bisphosphonates employed included alendronate, clodronate, pamidronate. The doses of these drugs and the methods of administration differed between the various centres. Oral alendronate was given continuously at a dose of 70 mg/week, to patients 1 and 7 for 24 months and 32 months respectively; oral clodronate was given 2 × 800 mg/day for 36, 12, 60 and 50 months to patients 2, 3, 4 and 6. Pamidronate, 90 mg/month was given intravenously to case 5. Zoledronic acid (4 mg) was given intravenously as a single infusion in cases 10, 20, 21; two infusions were given to cases 19, 22, 24; other cases received up to 6 intravenous infusions of zoledronic acid over a variable period of time. Protocols of drug administration varied between the centres but were generally correlated with tumour size and the response to treatment. The period of follow-up following treatment ranged from 36 to 64 months.

Primary, recurrent or metastatic GCTBs were regularly evaluated for disease progression. The effect of bisphosphonate treatment on tumour size was assessed radiologically by a variety of techniques including X-ray, MRI and CT. Clinical outcome was not assessed formally but when patients volunteered that their pain had decreased following treatment, this was noted. In patients with recurrent GCTBs in bone or soft tissue, it was also noted whether further recurrences occurred following therapy.

### Cell culture studies: Analysis of the effect of zolendronate on osteoclasts obtained from GCTB

Alpha minimum essential medium (αMEM) and fetal bovine serum (FBS) were purchased from Gibco Laboratories (Paisley, Scotland); αMEM containing 10% FBS, 100 U/ml penicillin, and 100 μg/ml streptomycin (MEM/FBS) was used for cell culture experiments. Zoledronic acid was obtained from Novartis (Basel, Switzerland).

Isolation of osteoclastic giant cells from GCTB cases was carried out as previously described [3]. Fresh tissue from four cases of GCTB, two (cases 11 and 12) of which were included among the clinical cases studied, was obtained at the time of surgery at the Nuffield Orthopaedic Centre, Oxford. GCTB tumour tissue was washed in sterile phosphate buffered saline (PBS). Fragments of tumour were curetted in αMEM/FBS and the cell suspension was added to dentine slices and glass coverslips in a 96-well plate (approximately 30 giant cells per well). After 2 hours incubation at 37°C in a humidified atmosphere of 5% CO_2 _and 95% air, the dentine slices and glass coverslips were washed in MEM/FBS to remove any non-adherent cells. Fresh MEM/FBS was then added and the cells were incubated in the presence and absence of zoledronic acid (4 × 10^-5 ^M). Cultures on dentine slices were maintained for up to 48 hours and including 24 hours at 37°C, then treated with 1 M ammonium hydroxide, washed in distilled water and ultrasonicated to remove adherent cells; these slices were then stained with 0.5% (W/v) toluidine blue to reveal areas of lacunar resorption and examined by light microscopy. The percentage surface area of lacunar resorption on each dentine slice was measured using image analysis software (Adobe Photoshop CS2, USA) as previously described [3].

## Results

### Effect of bisphosphonate treatment on primary, recurrent and metastatic GCTBs

Details of treatment, period of follow-up following therapy and radiological/clinical outcome are shown in Table 1. None of the patients reported complications related to bisphosphonate treatment.

**Figure 1:**
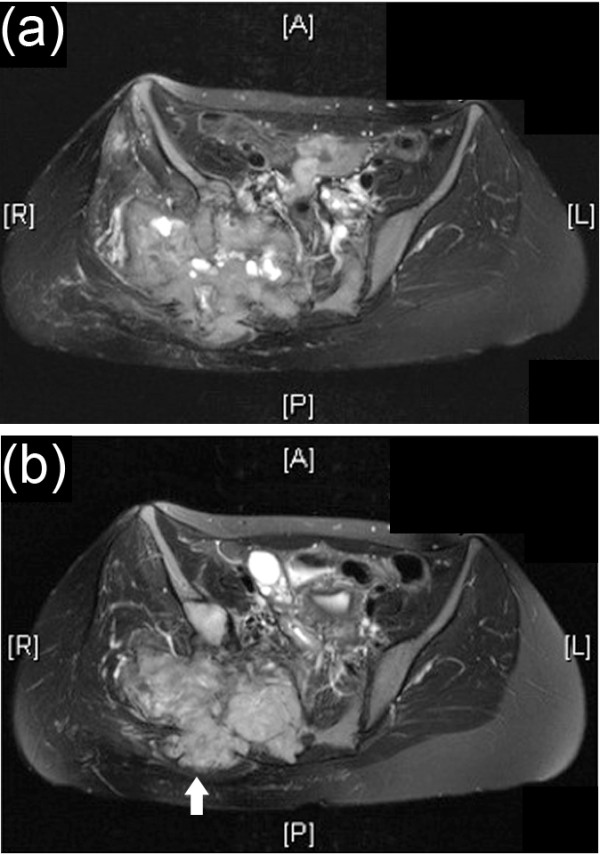
**MRI of GCT pre and post zoledronic acid treatment**. Axial T2- weighted (fat-suppressed) MRI of (a) pre and (b) 5 year post-zoledronic acid treatment of Case 12, an enlarging sacral/pelvic GCTB which shows stabilisation of tumour size. Following treatment there is some evidence of infilling of the lytic tumour (arrowed) following zoledronic acid treatment.

The majority of large primary sacral/pelvic GCTBs, three of which were deemed inoperable, did not increase in size following treatment. Tumours did not disappear or regress but remained stable in size. In two cases, increased calcification developed around and within the lesion (Figure 1). With the exception of one spinal and one sacral GCTB, both of which recurred following surgery, the other spinal and pelvic GCTB cases did not exhibit disease progression. Of the cases that recurred, one was a spinal GCTB which developed recurrence after incomplete excision 24 months after Zoledronic acid treatment (case 12); the other case was on Zoledronic acid treatment for only three months and died intra-operatively with extensive pelvic disease (case 23). Almost all sacral and pelvic tumours were also treated by embolisation and four cases received radiation therapy.

Seven patients received pre-operative or intra-operative Zoledronic acid for treatment of repeated recurrence of large GCTBs arising in the skeleton. Although several different types of bisphosphonate were employed (using different dosage and methods of administration), stabilisation of tumour size and some pain relief was noted. This beneficial effect was not restricted to the use of one type of aminobisphosphonate or method of administration, cases 1-7 receiving alendronate or clodronate orally, case 5 receiving Pamidronate and cases 8-25 receiving Zoledronic acid parenterally. Further recurrences did not occur after treatment in most cases and in two cases (cases 11 and 24), where two episodes of soft tissue recurrence had previously occurred, no further soft tissue recurrence developed.

Twelve of the cases developed lung metastases of GCTB. One of these cases had a multifocal GCTB with lung metastases. Two of the cases had metastases at the time of initial diagnosis (cases 11 and 13). In the other cases, metastases appeared following development of recurrent GCTBs. Metastases did not increase in size or number following bisphosphonate treatment as assessed by lung CT and chest X-ray. In one case (case 3) the CT findings were reported as showing a slight decrease in the size of lung metastases. No patient with metastatic disease developed further lung nodules whilst under treatment.

### Effect of Zoledronic acid on lacunar resorption by GCTB-derived osteoclasts

**Figure 2:**
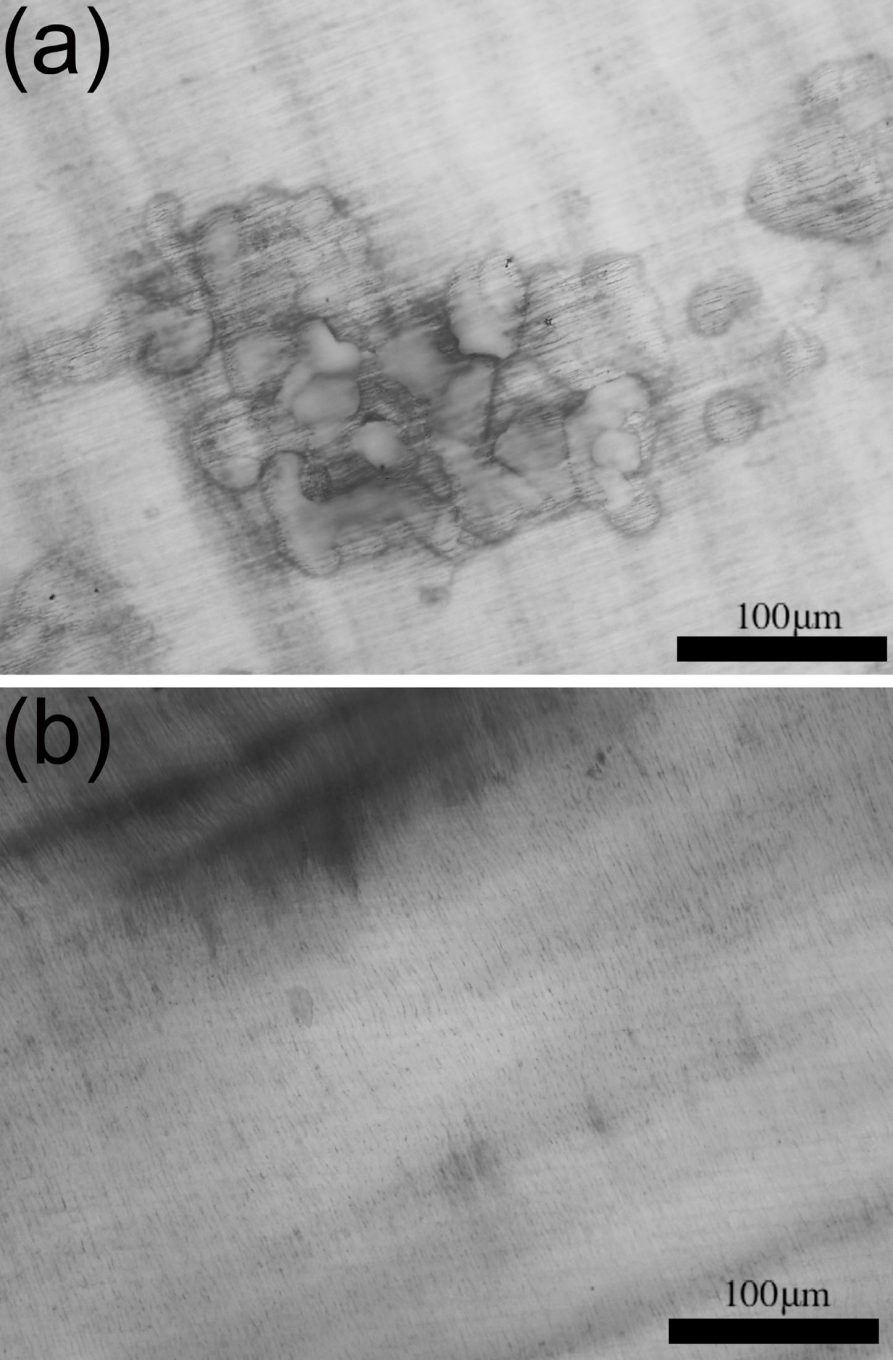
**GCT osteoclast resorption**. (a) control and (b) zoledronic acid-treated cultures of GCTB-derived osteoclasts on dentine slices, showing marked inhibition of lacunar resorption pit formation in zoledronic acid-treated cultures.

Multinucleated cells isolated from GCTB expressed the antigenic phenotype of osteoclasts, being TRAP and CD51 positive and CD14 negative [3,19]. The multinucleated giant cells were large and had abundant cytoplasm with broad pseudopodia. Lacunar bone resorption is the definitive functional criterion of an osteoclast and the GCTB-derived multinucleated cells were capable of carrying out lacunar resorption in short term culture. The addition of zoledronic acid (4 × 10^-5 ^M) to 24 hour cultures of GCTB-derived osteoclasts resulted in inhibition of lacunar resorption (Figure 2). The lacunar resorption in zoledronic acid-treated osteoclast cultures derived from two GCTB cases was 16% and 25% of that seen in untreated GCTB control osteoclast cultures and, in one case, a GCTB in which there was extensive aneurysmal bone cyst change, resorption activity was entirely abolished by zoledronic acid. Inhibition of resorption activity was not uniformly profound as one case showed only a 21% reduction in lacunar resorption relative to control osteoclast cultures.

## Discussion and Conclusions

GCTB commonly recurs after curettage and complete excision is often not possible due to the size and location of the tumour. This study reports the experience of four European centres which have employed aminobisphosphonates as a component of adjuvant treatment for problematic primary (inoperable), recurrent and metastatic GCTBs. Although results are difficult to assess as the problematic nature of the cases studied meant that results could not be compared with a single control group, and aminobisphosphonates were administered on an individual ad hoc basis, often in combination with other adjuvant therapies, they are of interest as they show that most tumours did not increase in size after bisphosphonate treatment and that control of tumour recurrence and pain was achieved in some cases.

Actively growing GCTBs are associated with extensive bone resorption. Bisphosphonate treatment is well-established in controlling osteolysis associated with skeletal metastasis. Paget's disease, osteoporosis, multiple myeloma [8,9], and bisphosphonates have also been used to control osteolysis occurring as a result of fibrous dysplasia and Langherans cell histiocytosis [12-14]. Two recent studies have reported the use of bisphosphonates in GCTB. Tse et al [16] studied 24 patients treated with either pamidronate or zoledronic acid given preoperatively and found that only one of 24 patients (4.2%) developed local recurrence following this treatment; this contrasted to the control group who had a recurrence rate of 30%. There is also a report on the use of intravenous bisphosphonate to control the growth of a giant cell tumour of the sacrum [15]. As in our series of cases, the patients receiving aminobisphosphonates for GCTB in these two reports, did not complain of significant treatment side effects.

Our results show that most inoperable primary sacral and pelvic GCTBs did not continue to increase in size after commencing oral or intravenous aminobisphosphonate treatment with some cases showing radiological evidence of bone formation and infilling of lytic lesions. Treatment also resulted in control of cases of persistently recurrent GCTB. Bisphosphonates are recognised to have an analgesic effect on bone tumours [20], and a decrease in pain was noted in some of the treated cases. This was most notable in case 13 where significant pain relief was achieved following zoledronic acid treatment of a large sacral tumour compressing the sciatic nerve; this tumour stabilized in size, developed a sclerotic border and showed infilling with bone; this patient is currently pain-free. The effect of bisphosphonates on metastatic lung lesions of GCTB was more difficult to evaluate as it is known that these lesions can remain stable in size for many years or even regress. However, it was noted that lung nodules of GCTB did not increase in size and no further nodules developed in the course of bisphosphonate treatment.

The tissue specific targeting of bisphosphonates to the mineral component in bone is extremely useful in controlling the growth of bone lesions. Bisphosphonates are non-toxic analogues of pyrophosphate which are known to inhibit osteoclast-mediated bone resorption. Binding of bisphosphonates to hydroxyapatite results in changes in the physico-chemical structure of the hydroxyapatite crystal [8-10]. Aminobisphosphonates act by inhibiting farnesyl diphosphate synthase, an enzyme in the mevalonate pathway. This results in inhibition of osteoclast formation from mononuclear phagocyte precursors, decreased osteoclast resorption due to effects on the cytoskeleton, vesicular trafficking and membrane ruffling, and increased osteoclast apoptosis [21-23]. It has been shown that bisphosphonate treatment induces apoptosis of GCTB stromal cells and osteoclasts [24-26]. An inhibitory effect of zoledronic acid on GCTB-derived osteoclast resorption in vitro was noted in this study; in three of the four cases osteoclast resorption was abolished or profoundly inhibited whereas in one case the decrease in resorption (21%) was more modest. This is in keeping with the findings of Lau et al [3], who also noted a pronounced but variable inhibitory response to zoledronic acid by GCTB-derived osteoclasts. This variation in the inhibitory effect of zoledronic acid on osteoclasts derived from different GCTBs may reflect differences in the therapeutic effect of bisphosphonates in our study.

GCTB contains numerous mature, functional osteoclasts. The rationale for using bisphosphonates to control the osteolysis associated with the growth of GCTBs is that these compounds reduce osteoclast numbers in bone and inhibit osteoclastic resorption. A similar rationale underlies treatment of GCTB with denosumab, an anti-RANKL antibody; this has been reported to result in clinical and radiological improvement in some cases [27]. Aminobisphosphonates have an established role in the treatment of tumour osteolysis associated with metastatic cancer and our results show that these drugs can achieve stabilisation of disease in problematic GCTB cases. The use of different bisphosphonates and protocols of treatment, as well as the effect of other adjuvant therapies, needs to be taken into consideration when evaluating the results of this study. None of the patients in our study reported significant side effects following bisphosphonate treatment but it should be borne in mind that long-term therapy with bisphosphonates can be associated with significant complications, notably osteonecrosis of the jaw [28].

Further studies to analyse the effect of aminobisphosphonates on GCTB would seem appropriate, particularly in the context of controlling the progressive bone destruction and enlargement of inoperable large primary GCTBs and problematic bone and soft tissue GCTB recurrences. It may be worthwhile examining whether combined denosumab and bisphosphonate treatment is more effective in controlling osteolysis or in alleviating bone pain; the latter can be assessed using standardised scores eg functional assessment of cancer therapy-bone pain [29]. Our study highlights the fact that there is no recognised protocol for aminobisphosphonate treatment of GCTB tumours in Europe (or elsewhere in the world to our knowledge), and that there is a need to establish standardised protocols not only for the administration of these drugs but also for definition of outcome measures that will permit a reliable assessment of treatment efficacy.

## Competing interests

The authors declare that they have no competing interests.

## Authors' contributions

MB, LC, JK, CG, CLMH, JW, NAA, PH and PP made substantial contributions to the conception and design of this study and the acquisition and analysis of patient data. RT and NAA carried out and analysed the in vitro studies. All authors consented to the implentation of the study, were involved in drafting and revising the manuscript and gave their approval of the final manuscript.

## Pre-publication history

The pre-publication history for this paper can be accessed here:

http://www.biomedcentral.com/1471-2407/10/462/prepub
